# Possible Mechanisms of Green Tea and Its Constituents against Cancer

**DOI:** 10.3390/molecules23092284

**Published:** 2018-09-07

**Authors:** Yohei Shirakami, Masahito Shimizu

**Affiliations:** 1 Department of Gastroenterology, Gifu University Graduate School of Medicine, 1-1 Yanagido, Gifu 501-1194, Japan; shimim-gif@umin.ac.jp; 2 Department of Informative Clinical Medicine, Gifu University Graduate School of Medicine, 1-1 Yanagido, Gifu 501-1194, Japan

**Keywords:** catechin, green tea, cancer, chemoprevention, receptor tyrosine kinase

## Abstract

A number of epidemiological, clinical, and experimental researches have indicated that administration of green tea appears to have anti-cancer activity. According to findings of laboratory cell culture studies, a diverse mechanism has been observed underlying the effects of green tea catechins against cancer. These mechanisms include anti-oxidant activity, cell cycle regulation, receptor tyrosine kinase pathway inhibition, immune system modulation, and epigenetic modification control. This review discusses the results of these studies to provide more insight into the effects of green tea administration on cancers observed to date in this research field.

## 1. Introduction

One of the most widely consumed beverages in the world, tea, has been taken since ancient times, with tea leaves originally used for medicine [1,2]. Produced from leaves of the plant *Camellia sinensis*, tea is usually categorized into three types—green, black, and oolong—due to leaf processing. Green tea is produced by drying and steaming tea leaves to prevent fermentation, while tea leaves for black or Oolong teas are totally or partially fermented, respectively [3,4]. Among teas, over 70% is black tea, which is commonly consumed in western countries, and approximately 20% is green tea consumed primarily in Asia and the Middle East [5]. Green tea has been most extensively investigated for its health advantages.

Tea contains polyphenols, which are crucial constituents, including catechins and flavonoids. Green tea possesses high quantities of green tea catechins (GTCs) in comparison with other types of tea [6]. Among the major catechins in green tea, such as (−)-epigallocatechin-3-gallate (EGCG), (−)-epicatechin-3-gallate (ECG), (−)-epigallocatechin (EGC), and (−)-epicatechin (EC), EGCG is the most plentiful being approximately 70% of the entire catechin constituent [7].

Currently, tea constituents, especially GTCs, have been the focus of scientists because of their possible preventive and therapeutic effects on chronic diseases, including cardiovascular disease, metabolic syndrome, and malignancy. GTCs have also been reported to exert beneficial actions against diabetes mellitus, stroke, Parkinson’s disease, and Alzheimer’s disease [8,9,10,11]. A number of epidemiological studies have shown that drinking green tea provides beneficial effects on human health and that tea consumption is associated with decreased incidence of various chronic diseases, including cancers, although the evidence is inconclusive [12,13,14,15]. Numerous cell culture and animal examinations have demonstrated that diverse actions exert the cancer preventive and therapeutic effects of EGCG as well as green tea. The mechanisms include stimulation of anti-oxidant activity [16,17], alteration of the cell-cycle [18] and DNA methyltransferase [19], and suppression of mitogen-activated protein kinase (MAPK) and receptor tyrosine kinase (RTK) pathways [20,21].

This review aims to provide more insight into the effects and mechanisms of tea catechins, especially GTCs, against cancer, and to discuss the current research investigating the effects of GTCs on cancer development and possible mechanisms underlying of how they act as anti-cancer agents.

## 2. Anti-Cancer Action of Green Tea and Its Contents

In laboratory examinations as well as chemically- or genetically-induced carcinogenesis models in rodents, tea constituents and GTCs have been shown to exert anti-cancer action. Researchers have examined the effects on various cancer types, including skin, lung, oral cavity, esophagus, stomach, small and large intestine, liver, pancreas, mammary gland, bladder, and prostate cancer [9,22,23,24]. The following sections will state the mechanisms which may underlie the anti-cancer activity of GTCs in several organ sites mentioned above.

Treatment of human small-cell lung cancer cells with EGCG was found to lead to reduced activity of telomerase and decreased caspase-3 and -9 activities [25]. Similarly, the proliferation of non-small cell lung cancer (NSCLC) cell was inhibited by EGCG treatment [26,27]. One of these was observed in cell lines that were both sensitive and resistant to erlotinib, a molecularly targeted agent for lung cancer treatment [26]. A study examining the efficacy of erlotinib and/or EGCG revealed that combination treatment contributed to significant inhibition of cell proliferation, colony formation, and growth of NSCLC xenografts in comparison to treatment with either agent alone [26]. Other studies have also demonstrated that the development of chemically-induced lung cancer in several rodent models was suppressed markedly by green tea supplementation [28,29,30,31,32]. The standardized polyphenol, named Polyphenon E (PolyE), contains EGCG, other catechins, and caffeine, extracted from green tea. Administration of PolyE markedly decreased incidence and number of lung adenocarcinoma, [28,29] and progression of lung adenoma into adenocarcinoma in mice. Gene expression changes in a chemically-induced mouse lung cancer model were further examined, identifying over 80 genes that were differentially expressed in tumors, but not in normal tissues, and were reversed by GTCs administration [29].

Previous studies indicated that signal transductions of cell-surface receptor pathways were inhibited by EGCG in MDA-MB-231 human breast cancer cells, contributing to the reduced production of vascular endothelial growth factor (VEGF) [33,34]. A relation of EGCG with VEGF was also investigated in another study, where cell viability and angiogenesis were inhibited, and apoptosis was induced by EGCG treatment via reduced VEGF expression in MDA-MB-231 cells [35]. A similar study demonstrated that treatment with green tea polyphenols or EGCG suppressed breast cancer cell proliferation via inhibition of cell growth and induction of apoptosis [36,37,38,39]. In chemically induced rat mammary tumorigenesis models, treatment with GTCs decreased the incidence, multiplicity, and volume of mammary tumors [40,41,42].

The effects of EGCG on colorectal cancer have also been closely investigated. As well as the study indicating that EGCG suppressed the growth of HT-29 human colon cancer cells [43], our research group reported that either EGCG or PolyE preferentially inhibited the growth of various human colorectal cancer cells, including SW480, SW837, HCT116, and HT29, compared to normal human colon cells [44]. The growth of xenografts inoculated with the SW837 cell was also markedly suppressed by EGCG administration [45]. In studies using animal models of colorectal carcinogenesis, green tea administration was found to suppress the development of aberrant crypt foci (ACF), pre-malignant lesions in colorectum [46,47]. In our study, it was shown that EGCG or PolyE supplementation inhibited inflammation-related colorectal carcinogenesis in mice induced by azoxymethane (AOM) plus dextran sodium sulfate (DSS) [48]. This experimental mouse study is considered a model for colorectal cancer caused by chronic intestinal inflammation that mimics inflammatory bowel diseases, such as ulcerative colitis and Crohn’s disease. In addition, our group also indicated that EGCG supplementation decreased the development of ACF and β-catenin-accumulated crypts (BCACs), an additional type of premalignant lesion in the colorectum, in male C57BL/KsJ-db/db mice, a genetically-engineered animal model to exhibit obesity and type 2 diabetes mellitus [49].

In LNCaP human prostate cancer cells, EGCG induced apoptosis via modulation of intrinsic and extrinsic pathways [50]. Studies employing xenograft of prostate cancer cells exhibited decreased xenograft tumor growth with the administration of green tea extracts or polyphenols [51,52,53]. In a study employing a transgenic adenocarcinoma of the mouse prostate (TRAMP) model, researchers observed that supplementation of the polyphenol extracted from green tea markedly suppressed the incidence of the prostate tumor [54]. Inhibitory effects of oral GTCs infusion on the formation of the prostate tumor were also observed in a similar mouse model [55].

## 3. Possible Mechanisms of Action of Green Tea Catechins against Cancer

**Figure 1:**
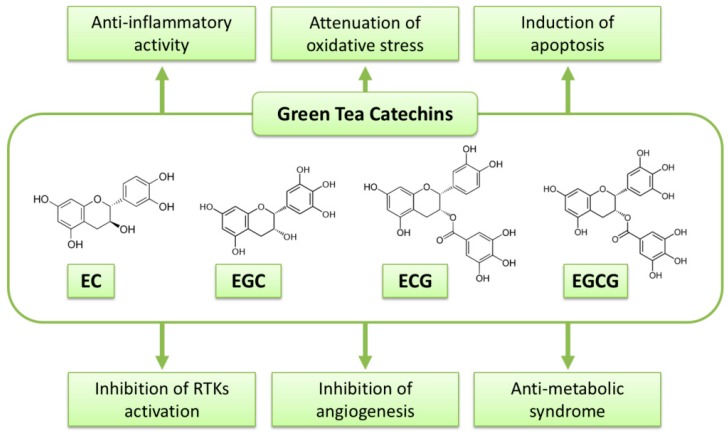
Possible mechanisms of green tea catechins against cancer.

EGCG, GTCs have been extensively investigated to reveal their anti-cancer mechanisms. A variety of mechanisms by which GTCs or EGCG display their biological activity in cancer cells and malignancies have been hypothesized. Several of them are stated in the following sections, and possible mechanisms of the action of GTCs against malignancy are summarized in Figure 1 and Table 1.

### 3.1. Anti-Oxidant and Pro-Oxidant Activities

The anti-oxidant ability of GTCs has been well demonstrated. Catechins possess anti-oxidant abilities by neutralizing free radicals. Among tea GTCs, ECG has the greatest potency as a radical scavenger, followed by EGCG, EGC, and EC [56,57]. A previous study indicated that GTCs exert a strong anti-oxidant function through quenching free radical species and chelating transition metals [16]. The anti-oxidant action is attributable to the presence of phenolic groups with sensitivity to oxidation which is able to generate quinine. The activity is further enhanced due to the trihydroxyl structure in the D-ring [16,58,59]. Although GTCs are believed to function as powerful radical scavengers, their anti-oxidant activities in animal models and human subjects are not conclusive.

The direct anti-oxidant activity of tea catechins was found primarily under conditions of increased oxidative stress, such as in patients with ulcerative colitis and hepatitis [16]. In an animal study, EGCG treatment attenuated lipid peroxidation and protein carbonylation in the livers of aged rats [60]. Interventional studies examining the anti-oxidant effects of green tea intake reported interesting results. Regular consumption of 600 to 1500 mL/day green tea increased the anti-oxidant ability in plasma and protected healthy subjects from DNA damage. The studies, however, demonstrated limited ex vivo and in vivo evidence that green tea intake provided anti-oxidant activity in cancer prevention [61], suggesting that green tea administration may display anti-cancer effects only in condition with excess oxidative stress.

On the other hand, the pro-oxidant activity of green tea polyphenols has also been reported and are well summarized in a previous paper by Lambert and Elias [16]. GTCs can generate reactive oxygen species (ROS), which are essential for the induction of apoptosis and lead to the inhibition of cancer cell growth [11,16]. Both anti- and pro-oxidant activities of GTCs are thought to be important against malignancy, especially for cancer prevention, and to play roles in different aspects of the oncogenic process [16].

### 3.2. Induction of Apoptosis and Cell Cycle Arrest

Apoptosis is considered a programmed cell death and is hypothesized to exert an important role in eliminating cancerous cells and to act as a protective mechanism against the development of malignancy [62]. Studies have shown that EGCG treatment induced apoptosis due to the generation of ROS and caspase-3 and -9 activations, leading to cell-cycle arrest at G1 phase via controlling expressions of cyclin D1, cdk4, and p21^CIP1^ [11,63]. In human head and neck squamous carcinoma (HNSCC) cell lines, EGCG treatment increased the proportion of G1 phase, decreased cyclin D1 protein levels, and increased p21^CIP1^ and p27^KIP1^ protein levels [64]. Other researchers reported that EGCG reduced Bcl-2 and Bcl-xL protein levels and increased Bax with caspase-3 activation [65]. In a human colon cancer cell, treatment with either EGCG or PolyE was observed to increase the ratio of cells in the G1 phase and to induce apoptosis. The treatment also decreased cyclin D1 and Bcl-xL proteins and increased caspase-3 and -9 activities [44].

In a colorectal carcinogenesis model employing obese and diabetic mice and carcinogen AOM, EGCG administration suppressed the development of pre-cancerous lesions in the colorectum and significantly decreased cyclin D1 levels in the colonic mucosa of mice [49]. The results suggest that green tea and the contents exert anti-cancer activity by regulating cell cycle arrest and inducing apoptosis through diverse mechanisms.

### 3.3. Inhibition of NK-κB and AP-1

A transcriptional factor nuclear factor-κB (NF-κB) is known to play a vital role in inhibiting apoptosis in cancer cells, leading to carcinogenesis [66]. Previous examinations demonstrated that EGCG treatment inhibited NF-κB activation in human HNSCC, breast cancer, and lung cancer cells [33,67]. When NF-κB is activated, it is translocated into the nucleus, which leads to diverse gene expression associated with carcinogenesis and tumor progression, including cellular transformation, proliferation, invasion, metastasis, radio-resistance, chemo-resistance, and inflammation. In human colon cancer and epidermoid carcinoma cells, NF-κB activity and its nuclear translocation were also inhibited by EGCG [18,68].

Another transcription factor activator protein-1 (AP-1) regulates gene expression levels related to apoptosis and cellular proliferation. It is considered that AP-1 promotes proliferation through up-regulation of cyclin D1 gene expression and down-regulation of tumor-suppressor genes, such as p53 and p21^CIP1^ [68]. EGCG was reported to suppress AP-1 activation and cell transformation and to inhibit Ras-activated AP-1 in a mouse epidermal cell line [69,70]. Studies by our research group in a human colon cancer cell line revealed that EGCG inhibited transcriptional activities of AP-1 and NF-κB promoters, as examined by reporter assay and that treatment with either EGCG or PolyE caused inhibition of AP-1 and NF-κB luciferase reporter activities [44,71]. These findings suggest that inhibiting NF-κB and AP-1 pathways is one of the important mechanism underlying the anti-cancer activity of GTCs.

### 3.4. Inhibition of Receptor Tyrosine Kinase Pathways

Previous studies have shown that receptor tyrosine kinases (RTKs) play pivotal roles in cellular proliferation and apoptosis and are proposed targets by GTCs for cancer prevention [72,73]. RTKs and their downstream signals, including the Ras/extracellular signal-regulated kinase (ERK) and phosphatidylinositol 3-kinase (PI3K)/Akt signaling pathways, regulate the expression levels of various target genes associated with proliferation and apoptosis [74,75]. Binding of cytokines and growth factors as specific ligands to the extracellular domain of RTKs activates intrinsic tyrosine kinase and induces phosphorylation of tyrosine residues, leading to the creation of docking sites for downstream targets [74,75]. In this manner, activation of cell-surface RTKs and downstream signaling pathways fulfill roles in the modulation of various essential processes.

Pre-cancerous or cancer cells frequently display inappropriate or constitutive activation of RTKs through mutation and over-expression of various genes [76,77]. The epidermal growth factor receptor (EGFR) is a member of the ErbB family of receptors, a subfamily of four closely related RTKs: EGFR (ErbB-1), human epidermal growth factor receptor (HER) 2/neu (ErbB-2), HER3 (ErbB-3), and HER4 (ErbB-4). Insulin-like growth factor-1 receptor (IGF-1R) and VEGF receptor (VEGFR) belong to a separate family of RTKs. Irregularities in some RTKs, especially EGFR, VEGFR2, and IGF-1R, are deeply associated with the properties of malignancy [76,77]. These findings indicate that RTKs, such as EGFR and HER2, are targets in the prevention and therapy of malignancies. In practice, RTK modulators are used for the treatment of various types of cancer, including lung, breast, stomach, and colon cancer [78].

It has been reported that GTCs affect RTKs in a beneficial manner. Several studies reported anti-cancer effects of EGCG on the VEGF/VEGFR axis. EGCG treatment suppressed VEGF production via inhibiting activation of signal transducer and activator of transcription (STAT)-3 and NF-κB in human HNSCC and breast cancer cells [33]. In another study, EGCG treatment inhibited phosphorylation of VEGFRs and induced apoptosis in lymphocytic leukemia cells [79]. Our research group also indicated that EGCG suppressed the growth of tumor xenografts generated from human colon cancer and hepatoma cells by down-regulating VEGFR2, Akt, and ERK activation and VEGF expression [45,80].

Focusing on IGF/IGF-1R signaling, in vitro treatment with EGCG reduced IGF-1 and the activated form of IGF-1R levels and increased IGFBP-3 in human hepatoma and colon cancer cells, indicating that EGCG exhibited inhibitory actions on the IGF/IGF-1R axis [81,82]. As described above, in the study using TRAMP mice, administration of green tea polyphenols in drinking water inhibited prostate cancer development and its metastatic lesions [54]. This treatment was considered to reduce IGF-1 levels and to recover IGF-binding protein-3 (IGFBP-3) levels through reduced PI3K and phosphorylated ERK and Akt levels [54,83]. Our research group also reported similar experimental results in which colorectal pre-malignant lesions were significantly suppressed by drinking water containing EGCG in a mouse colorectal carcinogenesis model [49].

Studies indicated that EGCG treatment inhibited EGFR and HER2 activation and decreased activation of their downstream signaling pathways in HNSCC, colorectal cancer, and breast cancer cell lines [33,34,44,64] as well as HER3 activation [71]. The research group also observed that EGCG and PolyE treatment decreased EGFR and HER2 phosphorylation and led to a subsequent decrease in ERK and Akt phosphorylation [44].

Due to these observations, RTKs are thought to be promising targets of EGCG for its anti-cancer activity. In particular, “lipid rafts”, known as detergent-insoluble plasma membrane domains, were considered to exert important roles for signal processing of RTKs. It was found that the lipid organization on the plasma membrane was altered by EGCG followed by EGFR internalization into endosomes, which prevented ligands from binding to EGFR [84,85]. The EGFR degradation following internalization was induced by phosphorylation of the receptor at serine 1046/1047, which is associated with EGCG-mediated p38 MAPK activation [86]. This effect of EGCG on EGFR degradation appears to account for ubiquitous action to modulate RTKs on lipid rafts.

### 3.5. Modulation of Immune System

The immune system functions to fight against unusual conditions or abnormal agents in the body to prevent diseases, including cancer [87]. Green tea has been reported to enhance humoral and cell-mediated immunity, resulting in decreased risk of several cancers [88]. Inflammation is considered as one of the immune system responses, but inappropriate inflammation frequently causes various diseases. EGCG is known to have a strong anti-inflammatory effect with therapeutic potential, and a large number of in vivo studies found that green tea polyphenols administration attenuated inflammation.

Among the examinations, one found that administration of green tea polyphenols reduced the level of tumor necrosis factor (TNF) induced by lipopolysaccharide [89]. Similarly, our research group demonstrated that EGCG and PolyE administration decreased levels of several inflammatory cytokines, including TNF, in the colorectal epithelium and suppressed inflammation-related carcinogenesis in a mouse colorectal cancer model [48]. These observations suggest that GTCs administration may possess a favorable efficacy on inflammatory disorders through anti-inflammatory activity and inhibiting NF-κB activation [22].

A tryptophan catabolic enzyme indoleamine 2,3-dioxygenase (IDO) is thought to suppress effector T cell immunity and to play a pivotal role in inducing immune tolerance [90]. In human oral cancer cell lines, EGCG was found to markedly inhibit the expression of IDO [91]. The effects of EGCG on IDO expression were examined, which revealed that EGCG markedly decreased expression levels of interferon (IFN)-γ-induced IDO and its enzymatic activity in colon cancer cells [92]. The same research group investigated the effects of an IDO inhibitor 1-methyltryptophan (1-MT) and EGCG on colorectal carcinogenesis. The study demonstrated that either 1-MT or EGCG significantly attenuated IDO activity in serum and suppressed IDO-overexpressing pre-neoplastic lesions in the colorectum of an AOM-induced rat colon cancer model [93]. The observations above indicate that EGCG appears to exert inhibitory effects on cancers by suppressing IDO expression and function, suggesting that IDO-inhibiting agents, including EGCG, have the potential for immunomodulation against malignancy.

### 3.6. Epigenetic Alteration

Epigenetics is the reversible heritable alterations of gene expression, which occur without DNA sequence alteration. These changes exert significant roles in the regulation of general gene expressions and contribute to cancer development due to affecting histone modification, altering chromatin structure, and regulating non-coding microRNA expression [94]. Epigenetic silencing of DNA-repair and tumor-suppressor genes, which usually results from gene hypermethylation in the early stages of cancer, is often related to various diseases [95].

EGCG has been reported to alter epigenetics in cancer cells through histone modification as well as DNA methylation [96,97]. It was reported that EGCG suppressed DNA methyltransferase activation, leading to cytosine-phosphate-guanine demethylation and to the subsequent restoration of silenced tumor-suppressor genes, including retinoic acid receptor-β (RARβ), p16^INK4a^, and O^6^-methylguanine-DNA methyltransferase [98]. Other in vitro examinations revealed that EGCG treatment promoted partial demethylation for reactivation of hypermethylated RARβ in breast cancer cells, as well as decreased methylation of telomerase reverse transcriptase promoter [94,99]. There have been, however, other studies in which EGCG treatment had no significant effects on epigenetic alteration, including demethylation or the restoration of methylation-silenced genes. In addition, the observations of in vivo studies investigating the effects of EGCG on the reversal of hypermethylation and reactivation of silenced genes were inconclusive [100,101].

### 3.7. Anti-Metabolic Syndrome Effects

Metabolic syndrome consists of obesity, high blood pressure, hyperglycemia, and dyslipidemia. GTCs have been reported to have preventive effects against such conditions and medical disorders [102]. It was demonstrated that dietary EGCG reduced body weight in mice with an increase of fecal lipids, attenuation of insulin resistance, and a decrease of plasma cholesterol. The anti-obesity effects of green tea were also shown in human intervention studies [103]. Recently, metabolic syndrome is recognized as a major risk factor for various types of cancer [104]. GTCs are considered to possibly ameliorate the condition of metabolic syndrome, which leads to prevention of carcinogenesis. This possibility is well documented and reviewed in previous publications [105,106,107].

## 4. Conclusions

Anti-cancer functions and the molecular mechanisms of GTCs, especially EGCG, have been found in various kinds of animal models and in vitro experiments using different types of cancer cells. In addition, findings in a number of epidemiological and interventional studies have indicated that green tea administration exhibits clinical relevance and significant effects in cancer chemoprevention [108].

Considering the observations in in vivo examinations and clinical investigations, the bioavailability of GTCs following consumption are critical factors [109]. The absorption, distribution, and metabolism of GTCs in humans should also be taken into account. EGCG concentrations for exerting biological effects in several cell culture experiments were much greater than those in the tissue and plasma detected in human trials and animal experiments [89]. In fact, typical tea consumption usually has catechins reach plasma levels only into the low micro-molar range [110]. Therefore, it still remains unclear whether the observations in in vitro studies with high concentrations of EGCG are able to be directly extrapolated to cancer chemoprevention in animals and humans. Recently, various forms of tea catechins, such as pills and capsules, have been investigated to obtain higher concentrations of tea catechins in the tissues and plasma and to enhance their bioavailability [111,112].

The present review summarizes the effects of GTCs against cancer mediated through diverse mechanisms, including anti-oxidant and anti-inflammation activities, immune and epigenetic modification, and RTKs inhibition. There also appear to be reported anti-cancer effects of GTCs other than the ones described in this review. The research area is quite broad, and researchers have not yet fully grasped their mechanisms. To explicate the molecular mechanisms and account for the discrepancy between laboratory examinations and clinical studies, researchers should keep investigating the effects of tea catechins on prevention and treatment of malignancies.

## Figures and Tables

**Table 1:** Possible anti-cancer mechanisms of green tea catechins and their references.

Mechanisms	References
Anti-oxidant activity	[16,60]
Pro-oxidant activity	[11,16]
Induction of apoptosis and cell cycle arrest	[11,44,49,63,64,65]
Inhibition of transcriptional factors	[18,33,44,67,68,69,70,71]
Inhibition of receptor tyrosine kinase pathways	[33,34,44,45,49,54,64,71,79,80,81,82,83,84,85,86]
Modulation of immune system	[22,88,89,91,92,93]
Epigenetic alteration	[94,96,97,98,99]
Anti-metabolic syndrome effects	[13,105,106,107]

## Abbreviations

ACF	aberrant crypt foci
AOM	azoxymethane
AP-1	activator protein-1
BCAC	β-catenin accumulated crypt
DSS	dextran sodium sulfate
EC	(−)-epicatechin
ECG	(−)-epicatechin-3-gallate
EGC	(−)-epigallocatechin
EGCG	(−)-epigallocatechin-3-gallate
EGFR	epidermal growth factor receptor
ERK	extracellular signal-regulated kinase
GTC	green tea catechin
HNSCC	head and neck squamous carcinoma
IDO	indoleamine 2,3-dioxygenase
IGF	insulin like growth factor
MAPK	mitogen-activated protein kinase
1-MT	1-methyltryptophan
NF-κB	nuclear factor-κB
NSCLC	non-small cell lung cancer
PI3K	phosphatidylinositol 3-kinase
PolyE	Polyphenon E
RARβ	retinoic acid receptor-β
ROS	reactive oxygen species
RTK	receptor tyrosine kinase
STAT	signal transducer and activator of transcription
TNF	tumor necrosis factor
TRAMP	transgenic adenocarcinoma of the mouse prostate
VEGF	vascular endothelial growth factor
